# Pandemic Preparedness and Pandemic Arrival – An Ethnographic Observation

**DOI:** 10.1017/S0021932025000240

**Published:** 2025-06-16

**Authors:** Foday Mamoud Kamara

**Affiliations:** Njala University, Sierra Leone

**Keywords:** Pandemic, Sierra Leone, Ethnography

The Pandemic Preparedness Project (PPP) was an attempt to understand community-level pandemic preparedness, primarily using a longitudinal observational research method. Anthropologists refer to this type of approach as ‘ethnography’. An ethnographic study is based on long-term residence, during which the researcher becomes well-known to the group being observed and habituated to their way of life. The knowledge, attitudes, and practices of the observed group are studied, contextualised within the researcher’s own grasp – as far as possible – of a social totality, sometimes referred to as a ‘life world’. Within the field of epidemiology, the aim of this approach is to understand what informants mean when they report or discuss bio-social facts regarding infection and risks of infection, describe symptoms of disease, or respond to interventions designed to reduce the spread of infectious disease. Sierra Leone has a good number of social scientists, but they have mainly been trained in approaches typically involving the use of questionnaires and numerical methods and are less familiar with ethnographic approaches. I was trained in Political Science and then Public Administration. Ethnography came as a bit of a shock, and these notes are intended to help my fellow researchers better understand what might be involved in taking an ethnographic approach, especially in the context of an epidemiological study intended to shed light on biosocial data. Ethnographically, one becomes acculturated by immersing oneself in the customs and traditions of the community, becoming involved in community undertakings such as the observance of burial protocols, joining them on their farms, and participating fully in community work. In bereaved homes, I would pay condolences to the family as a show of concern for them and the departed soul. When serious matters arose in the community, the town chief would invite me to be part of the jury to resolve the issue. Through this, the community started to recognise me and bestow confidence in me.

I started my ethnographic journey in July 2019 in G., a large Kpa Mende village in southern Sierra Leone. My initial experience with the community was somewhat daunting, both for them and for me, in the sense that many people were afraid of me. At that point, many referred to me as a spy in their communities, working for the government. Others assumed I was some kind of journalist who had come to observe them and would later disseminate my findings over social media. However, this changed over time due to my extended stay with them (over two years), during which I became acculturated as a typical Kpa Mende man. After some time, I was simply taken as one of them, which allowed them to unveil hidden facts about various occurrences. With my tape recorder, I soon became a familiar sight in G and its numerous satellite villages. Carrying out my participant observation studies no longer caused fear or comment. This happened about six months into the first year of my ethnographic journey. I skillfully acculturated myself by learning their culture through participant observation of “anything and everything”.

As is typical of an ethnographer, I inquired into and made observations of anything and everything. I made thorough observations of their daily activities and happenings in the community and took notice of any important personalities who visited the community. Afterward, I wrote down relevant facts about the community. I was concerned and focused on my daily records of happenings in the community. Being part of the community for years was an added advantage that significantly aided me in learning more about the community and noticing any unusual occurrences. I also observed the community on various fronts, such as their perceptions about preparedness, the 2014–2016 Ebola epidemic and how it was overcome, and I discussed numerous stresses faced during the rainy season. My observations extended to visits to individual farmers on their farms or gardens to learn about the varieties of crops grown and the type of soil in the area. I observed how the community disposed of dirt and engaged them in discussions about various responses such as hand washing, the use of face masks, and the observance of social distancing during the Covid-19 pandemic. This was because our pandemic preparedness project initially aimed at gaining ideas and skills from them on how to prepare against pandemics.

The arrival of Covid-19 automatically switched our focus to response. Once I was so familiar to the people that I had faded into the background, I started to pay particular attention to conversations or events that seemed relevant to pandemic preparedness. People had lived through the Ebola epidemic in 2014-16, though without experiencing any actual cases, and still talked about it. There had been numerous cases in neighboring chiefdoms, and people knew how serious a disease it was. They had also become familiar with testing for Ebola, and one case I documented shed light on local ideas about the connection between immorality and disease. This is described in one of the papers of this Special Issue, where I am a co-author, so it will not be repeated here, other than to remark that it can be offered as an example of what an ethnographic approach captures that eludes other methods.

I undertook this work for several months before the Covid-19 pandemic struck. During this period, I conducted lots of interviews with people on their daily living or livelihoods, their worries about the recently concluded Ebola outbreak, and on hygiene and sanitation (G. lacked toilets, and their river was a dumping ground and a known health hazard). My focus at that time was purely on pandemic preparedness. I was then in place to witness how people coped with a new threat to their health and livelihoods, and the ways in which their responses changed when the pandemic arrived in April 2020.

One of the topics I had studied quite extensively before Covid-19 arrived was the way in which chiefdom authorities mobilized the community to resist Ebola Virus Disease in 2014–15. This was by means of a chiefdom Ebola Task Force committee, which imposed (nationally mandated) by-laws on Ebola control (Kamara et al. 2022). People were very clear in their conversations that strict restrictions on local movements had allowed them to maintain a zero-case rate for Ebola infection throughout the 2014-15 outbreaks. By the time I talked to them and listened to their conversations, this had begun to harden into a doctrine – that adherence to by-laws protects against infection. There were no treatments for Ebola in 2014-15. Vaccines had not yet been released. So, the local reading was that by-laws controlled the disease. The role of sanitation – e.g., chlorine spraying – in destroying vital material was downplayed. In fact, chlorine spraying was held responsible for some supposed Ebola deaths. Biohazards were controlled by strict application of human law.

Ebola came at a time when everyone was baffled about how to actually handle the disease. Even the international communities were confused at the start of the epidemic. At the initial stage, a ban was placed on bush meat by the World Health Organization. Even the medical staff did not have the knowledge to fight the disease, coupled with the poor health status of the country. However, with the implementation of by-laws, many lives were saved. One of the saddest things about Ebola was that its chain of transmission was very fast and quick. For instance, the virus in the dead was still active and could infect the living (close family trying to offer a befitting burial for the deceased), which was why it was called in Mende “Bonda hengbei” (family sickness).

However, the outbreak of Covid-19 in 2020 changed our research focus from preparedness to response. Initially, there was a widespread understanding that the Ebola response, based on by-laws and quarantine, would also protect against Covid-19. Markets and places of work were closed, and social distancing and mask-wearing were imposed (Field notes, M. market). Unlike Ebola, the symptoms of Covid-19 were much harder to detect and less dramatic. It was a disease affecting mainly older people, and I undertook household censuses showing that older people were only a small portion of the total population. Also, the disease spreads in a different way – in the air, and not through contact with body fluid – so it was much harder to control than Ebola.

There were two problems, therefore: people could not ‘see’ the disease, and the benefits of strictly imposed quarantine were much less obvious since there were very few, if any, serious cases. If the disease ever swept through G., even I was not aware of it. I also followed up on various other topics. One was the impacts of market lockdowns, which were very severe and responsible for much food insecurity, as markets were closed and food prices increased. I, therefore, dwelled on this topic by thoroughly examining the background to the introduction of lockdowns globally, lockdowns in Sierra Leone (especially, of course, in G. and surrounding villages), and their impacts on traders, farmers, bike taxi riders, and livelihoods in general. The ambiguity that surrounds Covid-19 was due to the numerous social media news reports about the pandemic from family members living abroad, stating that it is not real. The social media bloggers created doubts in the fight, creating more difficulty for the government. In the case of Sierra Leone, Liberia, and Guinea, Ebola left a marked impact in terms of death. The death rate from Covid-19 was a bit lower compared to some European countries that did not experience Ebola. In Sierra Leone, cases of Covid were lower, as well as deaths from Covid-19. The Chiefdom continued with its positive boasts of a zero-case record on Ebola. This was because the chiefdom Task Force was vigilant, and the continued trust in by-laws helped to prevent Ebola in the entire chiefdom. Indeed, all those fake social media messages made many people less effective in fighting the pandemic.

## Lockdown In Sierra Leone

Like in many other countries, Sierra Leone implemented lockdowns as a measure to fight the virus. Lockdown, according to the government’s National Coronavirus Emergency Response Centre (NaCOVERC), was a way to suppress the spread of the virus and involved curtailed movement of people, contact tracing, and surveillance. Through the Ministry of Health, NaCOVERC implemented a three-day lockdown (May 3, 2020, to May 5, 2020) in Freetown, a second longer lockdown in Freetown to cope with a second wave of the virus (June 21, 2021, to July 10, 2021), and the announcement of a night curfew for the entire country, as well as an inter-district lockdown. It is worth noting that, due to a lack of capacity for mass testing, there is no real evidence concerning the impact of these lockdowns.

In the fieldwork village, the national lockdown worked in line with local by-laws maintained from Ebola to further restrict movement and impose social distancing. Earlier Ebola experience was recapitulated when the Paramount Chief declared that, because of a recorded case and the chiefdom’s closeness with other chiefdoms, “I need to protect and fortify my own side of the border with the above chiefdoms so that those on the run cannot infect my people.” During Ebola, the Paramount Chief had been quick to consult with his chiefdom committee members to form a task force in his chiefdom to fight the Ebola threat. This idea was embraced by the task force and the chiefdom people. The committee made a series of by-laws to be supervised by the town chief of every village, controlling movement in and out of all villages, how strangers should be received, and what should be done when a stranger enters any village. These arrangements were seen to be successful in preventing Ebola from entering the chiefdom.

Similarly, with Covid-19, the Paramount Chief traveled to all his villages on a motorbike, informing villagers about the severity of Covid-19 and that people should be vigilant and stay in their villages. I recorded him stating, “What is worrisome now is the Yele case (in Bonkolenken chiefdom). If we don’t intensify the inter-village lockdown, infected people will enter our chiefdom.” His reaction was understandable in relation to the earlier Ebola threat. But lockdown meant to curb or prevent Covid-19 infection was somehow more disturbing to the locals. This was especially true of the lockdown instituted by the paramount chief, preventing inter-village movement for trade and other essential activities. Lots of traders, farmers, and motorcycle taxi riders expressed frustration at the loss of work, though the measures were meant to prevent the rapid spread of the virus.

The council and the paramount chief also decided to form a Task Force in all towns and villages of the chiefdom, where the committee members would include section chiefs, town chiefs, youth leaders, women leaders, etc., to help in the fight against Ebola, since it was a moving trend. The committee then made and endorsed chiefdom by-laws in addition to the national by-laws, such as the ban on daily bases, moving from one village to the other, a ban on visits from family members, and a total ban on visits from strangers (even if an indigene of the chiefdom) from entering any of the villages in the chiefdom. Anyone wanting to enter the chiefdom would be classified as strangers until the fight against Ebola was over. With these measures, the chiefdom was able to record zero cases of Ebola and Covid-19. The Task Force members were present in all parts of the chiefdom, mounted check points to check on those entering the country, and at the same time reject and return anyone from outside until the battle against Ebola was over.

## Impacts of Lockdown

After Covid-19 first reached Sierra Leone in January 2020, and the implementation of strict measures to prevent its spread across the country, lots of essential, everyday activities in farming and trade were halted. People worried about their fate and wondered if they could survive the disease, thinking about the Ebola outbreak. Many of my informants were bothered more by the many restrictions attached to the pandemic than the disease itself. One man told me, “I used to cultivate a large [upland] farm, engaged in swamp work and made vegetable gardens. But the pronouncement of Covid-19 paralyzed all my efforts in 2020.” He then added: “I’m afraid, even your presence has made me afraid; you are a journalist and have come to observe us and later send our information to the government. I don’t think I will talk to you. I’m totally discouraged about this pandemic.” But it was the confusion about how severe the epidemic would be that caused him the most difficulty: “I could not cultivate in 2020, my family suffered on the grounds that I was worried and confused about the pandemic and did not know when it would end.” Ebola was different; it was a terrifying experience, but because of a large international response, and the evident visibility of the disease, it was controlled in weeks. But Covid-19 dragged on for two years and more, and never reached any evident crisis point. Nor did it respond to all-out efforts at control. It was elusive, and people could barely distinguish it from other infections passing around.

Another man confirmed to me that the 2020 farming year had been very bad. “I don’t know whether it is because of the pandemic that farms did not do well, or due to the weather and the hesitating minds of the farmers about prevailing health conditions in the country. For two years, from the end of 2019 to 2021, as farmers, we were faced with a lot of embarrassing failures in our harvests. Some people denied it had anything to do with the epidemic and attributed it to bad luck”.

Mask wearing was a surprising success. People saw it as beneficial in protecting others, as well as themselves. Peasant food-producing communities are generally good at mutuality; they typically share a lot. Motorcycle taxi riders quickly realized that masks were good protection against dust and have continued to wear them even as the pandemic has receded. Initially, people were reluctant to wear masks. However, when they realized the benefit and their role in breaking the chain of transmission, masks became commonplace. People now wear them while walking on the street against dust and violent wind, mainly bike riders, and many use them to prevent inhaling infected air from the atmosphere, e.g., tuberculosis or other airborne diseases.

Masks became utility clothing. People see them as more useful and protective against airborne infections. It was framed in such a way that wearing a mask would prevent infection. Abiding by by-laws and wearing masks conveyed a feeling of concern for the family, community, and the country at large. The reason for wearing them was to curtail the transmission rate. Sharing of love, concern, and mutuality were some of the actions that helped to defeat the Ebola and Covid-19 pandemics. Community action was paramount and played a pivotal role, as seen in the Kamajei Task Force. The chiefdom as a whole thinks of themselves as one and members strong in relationship in the fight of pandemic rather as individual. One piece of evidence lies with the continuous wearing of masks against airborne diseases and many other hazards within the atmosphere. When people started using masks, the infection rate reduced drastically.

Later, vaccines arrived, and administration started on March 22, 2021. Vaccines were given to health workers, security personnel, military, prison officers, and teachers aged over 40 when supplies were limited. Later, when more arrived, vaccines were extended to the public. I spent time following up with informants about their changing ideas about vaccines. Initial resistance was linked to misinformation. I followed one chief, who had earlier told me he would never take the vaccine. Later, I found that he had changed his mind and had taken it. He explained that he thought that by-laws were the best protection from disease but later decided he should get vaccinated since he was a figure of authority in the community. If he did not set an example, then other people might not follow the by-laws, which he saw as the real way of protecting against the disease. These changing attitudes to vaccination show that pandemics are moving events, and participant observation is needed to monitor often rapidly changing attitudes, understandings, and responses.

My advice to the government, policymakers, and donors will be to change the approach to intervention in the fight against pandemics. Attention should be drawn to the less privileged communities that are vulnerable and hard to reach, and a bottom-up approach should be used in addressing pandemics. Countries should strengthen their health systems and work along with international best practices in the fight against pandemics. When approaches start with the grassroot communities, more facts can be revealed as compared to the top-down approach, where laws, policies, and expertise flow from the top to the villages. Building the hub of preparedness starts with the bottom that housed the vulnerable and less privileged.

I admired and entered the field of research with the fact that I have passion and love for it because it is knowledge-based. Research always takes one from the unknown to the known. Ethnographers are like observers placed in a particular community to thoroughly observe and write down facts from the community. My journey into ethnography has exposed me extensively in the field of research. It has enabled me to interact with various stakeholders at the policy level on how to fight pandemics. I was privileged to interface with various response teams regionally and nationally in the country. While in the village, I contributed immensely to educating my community on by-laws announced by the government. While in the fight against Covid-19, I came to realize that pandemics are moving trends and their fight should be fluid and continual.

As an ethnographer, I lived in a particular village for years and engaged in participant observation and later wrote down relevant points daily. Unlike other surveys, researchers stay out of the community and visit on a timely basis to get information about the community. I prefer ethnography for research because it focuses on the community and its people. By staying with the community, more facts can be revealed. My advice to anyone who would like to be a researcher on pandemic preparedness is that the grassroots or vulnerable communities must be researched so that information collected can be reflective on the ground. No ethnographic research can succeed without staying with the community for years as I did. Researchers, therefore, should not wholly and solely depend on secondary data but rather be in constant touch, if not stay, in the community (making you more anthropological), thereby getting the raw facts of your research from self-observation.

## Conclusion

This ethnographic study highlights the critical need for adaptive, community-centered approaches to public health crises. The initial reliance on by-laws and quarantine measures, modeled after the perceived success of Ebola containment, proved insufficient due to fundamental differences in disease transmission and community perceptions. Where the visible and rapidly devastating nature of Ebola fostered widespread compliance with strict regulations, the less dramatic and more ambiguous threat of COVID-19, coupled with its prolonged disruption of livelihoods, led to confusion and resistance.

The imposition of blanket lockdowns, for example, severely impacted local markets and agricultural activities, exacerbating food insecurity and eroding trust in public health interventions. As one informant lamented, the COVID-19 restrictions “paralyzed all my efforts in 2020,” underscoring the unintended consequences of top-down approaches that fail to account for local economic realities and coping mechanisms.

These findings underscore the necessity of a “bottom-up” approach to pandemic preparedness and response. Such an approach necessitates engaging directly with communities to understand their specific needs, concerns, and existing resources. This would allow for targeted interventions that minimize economic disruption while effectively addressing transmission risks. For instance, supporting local markets in implementing safer practices, rather than enforcing complete closures, and developing culturally appropriate messaging in collaboration with community leaders would foster trust and promote sustainable behaviour change.

Moreover, the evolving nature of the pandemic demands a “fluid” approach that adapts to new information, changing attitudes, and emerging challenges. The initial resistance to vaccination, as illustrated by the shifting perspectives of the local chief, exemplifies this need for adaptability. The chief’s initial reliance on by-laws as the primary protection against disease, later tempered by his recognition of vaccination as a means of promoting community adherence to public health guidelines, demonstrates the importance of continuous monitoring and flexible strategies. A fluid approach involves ongoing assessment of community perceptions, targeted communication to address misinformation, and adjustments to intervention strategies based on real-time feedback.

Crucially, this ethnographic study revealed insights that would have been inaccessible through other methodologies. While surveys might capture surface-level attitudes, ethnographic observation provides the nuanced context and deeper understanding of social dynamics that shape community responses to pandemics. The chief’s initial vaccine hesitancy, for example, was not simply a matter of individual skepticism but was rooted in a broader belief system that prioritized local by-laws as the most effective means of disease control. By understanding these underlying beliefs, public health interventions can be tailored to align with existing cultural frameworks and build trust within the community.

In conclusion, effective pandemic preparedness and response require a shift away from top-down, one-size-fits-all approaches towards adaptive, community-centered interventions. By prioritizing local knowledge, building trust through participatory engagement, and continuously monitoring community perceptions, policymakers can create more resilient and effective public health systems that protect vulnerable populations and foster sustainable behavior change.

